# Identification of a QTL region for tomato brown rugose fruit virus resistance in *Solanum pimpinellifolium*

**DOI:** 10.1007/s00122-025-04974-0

**Published:** 2025-07-22

**Authors:** Namrata Jaiswal, Bazgha Zia, Bidisha Chanda, Andrea Gilliard, Ainong Shi, Kai-Shu Ling

**Affiliations:** 1 United States Department of Agriculture-Agricultural Research Service, U.S. Vegetable Laboratory, Charleston, SC 29414 USA; 2 Department of Horticulture, University of Arkansas, Fayetteville, AR 72701 USA; 3 Present Address: United States Department of Agriculture-Agricultural Research Service, Crop Production and Pest Control Research, West Lafayette, IN 47907 USA; 4 Present Address: Sakata Seed America, Salinas, CA 93906 USA

## Abstract

Tomato (*Solanum lycopersicum* L.), one of the most widely grown vegetables in the world, has been seriously impacted in the past decade by the emerging tomato brown rugose fruit virus (ToBRFV). ToBRFV is a seed-borne tobamovirus, with ability to overcome the commonly used *Tm*-2^2^ resistance gene in tomato. The objective of this study was to conduct quantitative trait locus (QTL) mapping and identify single-nucleotide polymorphism (SNP) markers associated with ToBRFV resistance in tomato. Two F_2_ populations were used for QTL mapping: One derived from a cross between *S. pimpinellifolium* USVL333 (PI 390718) × USVL332 (PI 390717) and another from ‘Moneymaker’ × USVL332 (PI 390717), with population sizes of 195 and 79 plants, respectively. The resistance trait was derived from the *S. pimpinellifolium* accession USVL332 (PI 390717). A major QTL for ToBRFV resistance was identified on chromosome 11 (SL4.0ch11), with the peak located at approximately 46.84 Mbp. This QTL spans a 22-kb interval between 46,825,788 bp and 46,847,421 bp, as determined through both genome-wide association study (GWAS) and QTL linkage mapping. Three SNP markers, SL4.0ch11_46825788, SL4.0ch11_46847421, and SL4.0ch11_46850215, demonstrated the most significant association with high LOD values (LOD = 13 in the Blink model) in GWAS analysis. In this genomic region, two disease resistance gene analogs, Solyc11g062150 (TIR-NBS-LRR resistance protein, Toll-Interleukin receptor) and Solyc11g062180 (disease resistance protein, leucine-rich repeat), were identified, which may serve as candidates for ToBRFV resistance. The QTL identified in this study could be valuable for plant breeders in facilitating tomato breeding with ToBRFV resistance.

**Supplementary Information:**

The online version contains supplementary material available at 10.1007/s00122-025-04974-0.

## Introduction

Tomato (*Solanum lycopersicum* L.), one of the most important vegetable crops globally, has an annual farm gate value exceeding $2 billion in the United States (U.S.) (FAO Statistics 2021). California leads in tomato production for processing, while Florida dominates fresh-market tomato production. Additionally, the U.S. imports significant quantities of fresh tomatoes, primarily from Mexico and Canada, which are often grown in greenhouses (Guan et al. 2018). Domestic greenhouse tomato production in the U.S. is also expanding but remains insufficient to meet the strong demand (Guan et al. 2018). However, the intensive production practice and controlled greenhouse environment in greenhouse tomato productions have facilitated the emergence of several seed-borne and mechanically transmitted viruses, posing significant challenges to the tomato productions worldwide (Rivarez et al. 2021).

Tomato brown rugose fruit virus (ToBRFV), an emerging tobamovirus, presents a severe threat to global tomato production (Salem et al., 2023; Zhang et al. 2022). First identified in Jordan and Israel in 2014–2015 (Luria et al. 2017; Salem et al. 2016), ToBRFV has since spread to more than 40 countries worldwide (EPPO 2025). The rapid spread of ToBRFV has resulted in a global pandemic (Salem et al., 2023; Zhang et al. 2022). In the U.S., ToBRFV was first detected in greenhouse-grown tomatoes in California in 2018 (Ling et al. 2019). To mitigate its spread, the USDA-APHIS issued a Federal Order in 2019, later amended in 2024, requiring inspections of imported tomato and pepper seeds and propagating materials (USDA-APHIS 2024).

The global outbreaks of ToBRFV have been attributed to its seed-borne nature, resistance-breaking ability against resistance genes (*Tm-1*, *Tm-2*, and *Tm-2*^*2*^), and increased international seed and produce trade (Chanda et al. 2021b; Luria et al. 2017; Maayan et al. 2018). For over 60 years, the *Tm-2*^*2*^ gene (PI 128650) has been used to confer resistance to other tobamoviruses such as tomato mosaic virus (ToMV) (Alexander 1963; Carr 2024; Spiegelman and Dinesh-Kumar 2023). However, ToBRFV’s resistance-breaking capabilities have necessitated significant efforts to identify new genetic resistance sources, including work by public institutions (Kabas et al. 2022; Jaiswal et al. 2024; Jewehan et al. 2022a, 2022b; Zinger et al. 2021) and private companies through patent filings (Ashkenazi et al. 2018; Hamelink et al. 2019; Millenaar et al. 2021; Ykema et al. 2020). Several quantitative trait loci (QTLs) associated with ToBRFV resistance have been identified. These include regions on chromosomes 6, 9, and 11 in *S. lycopersicum* (Ashkenazi et al. 2018), chromosomes 2 and 11 (Zinger et al. 2021), and chromosome 11 (Millenaar et al. 2021). To our knowledge, there is still no available information on QTL analysis for ToBRFV-resistant *S. pimpinellifolium* which was recently identified (Jaiswal et al. 2024).

To support tomato breeding, there is an urgent need to identify molecular markers associated with ToBRFV resistance from the newly identified genetic resources. Our earlier research identified a resistant line of *S. pimpinellifolium* (PI 390717) that exhibits asymptomatic responses and low to undetectable virus titers (Jaiswal et al. 2024). The objective of this study was to identify QTLs associated with ToBRFV resistance in *S. pimpinellifolium*. Using two F_2_ populations derived from interspecific (*S. pimpinellifolium* × *S. lycopersicum*) and intraspecific (*S. pimpinellifolium* × *S. pimpinellifolium*) crosses, we identified a major QTL on chromosome 11 in *S. pimpinellifolium* PI 390717*.* Associated single-nucleotide polymorphisms (SNPs) located within a narrow region on chromosome 11 have the potential to serve as molecular markers for marker-assisted selection in tomato breeding programs.

## Materials and methods

### Plant materials and F_2_ genetic population development

To study the genetic inheritance of ToBRFV resistance in *S. pimpinellifolium* USVL332 (derived from PI 390717), two populations were developed by crossing the resistant *S. pimpinellifolium* parent USVL332 with two susceptible parents: *S. lycopersicum* ‘Moneymaker’ and *S. pimpinellifolium* USVL333 (derived from PI 390718). These crosses produced F_1_ seeds, which were then self-pollinated to generate F_2_ populations. The resulting F_2_ populations included 205 individuals from an intraspecies cross of two *S. pimpinellifolium* parents, USVL333 x USVL332 (designated as UU), and 80 individuals from an interspecies cross between ‘Moneymaker’ and USVL332 (designated as MU). These populations were used for phenotyping ToBRFV resistance and genotyping through whole-genome resequencing using NovaSeq to identify QTLs associated with ToBRFV resistance in *S. pimpinellifolium* USVL332 (PI 390717).

### TOBRFV resistance evaluation

The phenotypic evaluation for ToBRFV resistance was conducted in a containment greenhouse at the U.S. Vegetable Laboratory in Charleston, SC, USA, using the ToBRFV-US isolate CA18-01 (GenBank accession no. MT002973) (Chanda et al., 2020; Ling et al. 2019). Virus inoculum preparation and mechanical inoculation of test plants followed the standard protocol described by Chanda et al. (2021b).

The developed populations were phenotyped based on symptom observation and serological tests to assess virus titers in the test plants. Symptoms were scored according to a disease severity index on a scale from 0 to 5. Class 0 represented asymptomatic systemic leaves; class 1, mild mosaic on systemic leaves; class 2, mosaic on systemic leaves; class 3, mosaic with leaf deformation; class 4, severe mosaic and mottling with leaf deformation; and class 5, severe mottling, leaf deformation, and string-like leaves (Jaiswal et al. 2024).

In addition to symptom-based phenotyping, serological testing (enzyme-linked immunosorbent assay, ELISA) for ToBRFV was performed according to the manufacturer’s instructions (Agdia, Elkhart, IN, USA). Plants classified as asymptomatic (class 0) with low or non-detectable virus titers were considered resistant (R). Plants with disease severity scores of 1 or 2 were considered moderately susceptible (MS), while those with scores of 3–5 were classified as susceptible (S).

### DNA extraction, sequencing, and SNP genotyping

Genomic DNA was extracted from freshly collected young leaves of each inoculated plant in two F_2_ populations using the DNeasy® Plant Mini Kit (Qiagen, Germantown, MD). Emerging trifoliate leaves were collected from each of the three parents and 285 F_2_ individuals. DNA concentration was adjusted to 10 µg/mL using a Nanophotometer® P-class (Implen, Westlake Village, CA).

The 285 F_2_ samples (205 UU and 80 MU), along with three parents, were subjected to whole-genome resequencing (WGR) with 2 × tomato genome size coverage (~ 2 Gb sequencing data) at the Texas A&M University’s Genomics and Bioinformatics Center. DNA sequence libraries were prepared using the PerkinElmer NEXTFLEX Rapid XP Kit protocol. Tomato samples were sequenced on an Illumina 6000 system with NovaSeq S4 flow cell and Xp workflow using the 2 × 150 bp recipe. FASTQ files were processed using the Illumina Dynamic Read Analysis for Genomics (DRAGEN) Bio-IT processor, and variant calling (SNP and insertions/deletions) was conducted using the DRAGEN pipeline (v3.8.4) to map sequence reads to the *Solanum lycopersicum* ITAG4.0 reference genome (https://data.jgi.doe.gov/refine-download/phytozome?organism=Slycopersicum&expanded=691) (Hosmani et al. 2019).

A total of 5,160,657 SNPs were identified across the 12 chromosomes in the two tested F_2_ populations. A chi-square test was performed on all 5,160,657 SNPs obtained from DNA sequencing using a Visual Basic (VB) script in Microsoft Excel. SNPs fitting a 1:1 segregation ratio for homozygous alleles (*P*-value > 0.01) and showing allele differences between the resistant parent (‘USV332’) and the two susceptible parents (‘Moneymaker’ and ‘USVL333’) were retained for further analysis. SNPs with more than 5% missing data were filtered out. After filtering, 440,962 SNPs remained.

A single-marker regression (SMR) analysis of these SNPs identified a QTL on chromosome 11. To reduce the dataset, 10,000 SNPs were randomly selected from each chromosome, except chromosome 11, where 26,357 SNPs were retained. Ultimately, 136,357 high-quality SNPs were used for further analysis in this study (Supplementary Figure S1). The SNP dataset for the tested population has been published in a public database at https://doi.org/10.6084/m9.figshare.25345042.v1.

### Association analysis

A genome-wide association study (GWAS) was performed using the 136,357 SNPs across the 274 F_2_ individuals. We employed three different statistical models—single-marker regression (SMR), general linear model (GLM), and mixed linear model (MLM)—to perform GWAS analysis in TASSEL 5 (Bradbury et al. 2007). Additionally, GWAS analysis was conducted using GAPIT 3 (Genomic Association and Prediction Integrated Tool version 3) with four methods: standard mixed linear model (MLM), multiple loci mixed model (MLMM), fixed and random model circulating probability unification (FarmCPU), and Bayesian-information and linkage-disequilibrium iteratively nested keyway (BLINK). The principal component analysis (PCA) parameter was set to 3 (Wang and Zhan 2021; https://zzlab.net/GAPIT/index.html; https://github.com/jiabowang/GAPIT3).

Through extensive analyses using multiple models in TASSEL 5 and GAPIT 3 programs, we intended to identify reliable and stable ToBRFV resistance-associated SNP markers, candidate genes, and QTL regions in the two F_2_ populations. Using Bonferroni correction with a *P*-value at α = 0.05, calculated as 0.05 / number of SNPs, significant thresholds of associations were determined (López-Hernández and Cortés 2019). A logarithm of odds (LOD) of 6.44 was used as the significance threshold based on the 136,357 SNPs analyzed in this study. Additionally, a *t*-test was performed on all 136,357 SNPs using Visual Basic for Applications (VBA) in Microsoft Excel 2016.

### Genetic mapping and QTL analysis

Linkage maps for the two F_2_ populations were constructed using JoinMap 4 (Van Ooijen 2006) and MSTmap (Wu et al. 2008; http://mstmap.org/). QTL mapping was performed using three different statistical methods: single-marker regression (SMR), single-trait Bayesian interval mapping (BIM), and single-trait multiple interval mapping (SMIM), implemented in QGene (Joehanes et al. 2008).

### Candidate gene identification/detection

Candidate genes were searched within the 327 kb confidence interval of the QTL region on the *Solanum lycopersicum* ITAG4.0 reference genome assembly (https://data.jgi.doe.gov/refine-download/phytozome?organism=Slycopersicum&expanded=691). Resistance gene analogs located within or flanking the QTL were considered potential candidate genes for ToBRFV resistance.

## Results

### Resistance response to ToBRFV

**Fig. 1:**
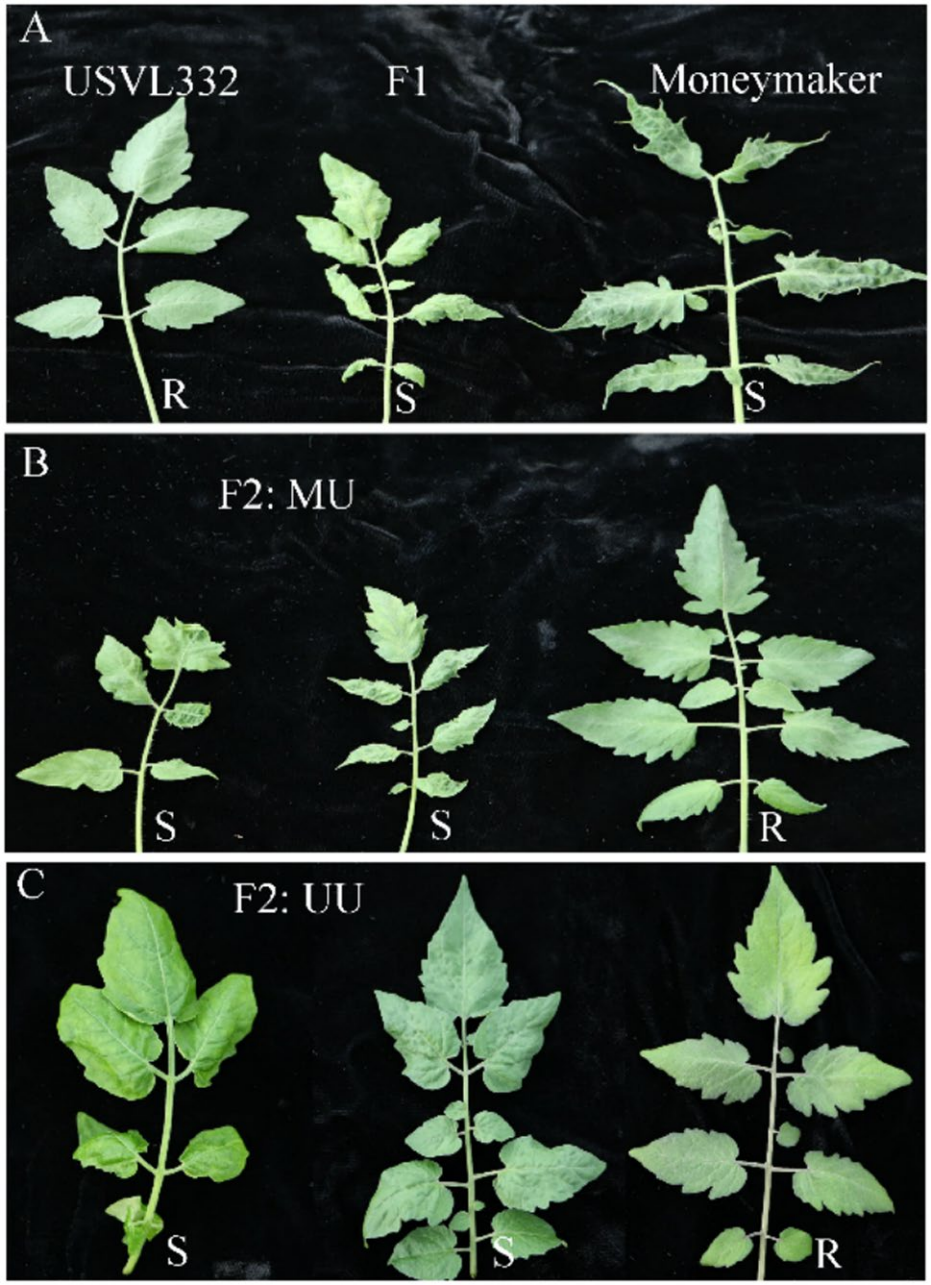
Representative phenotypes of genetic populations generated from their respective parents, F_1_ and F_2_ for resistance to tomato brown rugose fruit virus. **A** Phenotypic expression of the resistance line (*S. pimpinellifolium ‘*USVL332’) and the susceptible parent (*S. lycopersicum* ‘Moneymaker’) and their F_1_ plants upon inoculation with ToBRFV US isolate (CA18-01); **B** Representative phenotypes of a F_2_ MU population generated between ‘Moneymaker’ and ‘USVL332’ with mosaic, mottling, and leaf deformation (S) and normal healthy-looking leaves (R). **C** Representative phenotypes of a F_2_ UU population generated between ‘USVL333’ and ‘USVL332’ with mosaic and mottling (S) and normal asymptomatic leaves (R)

**Fig. 2:**
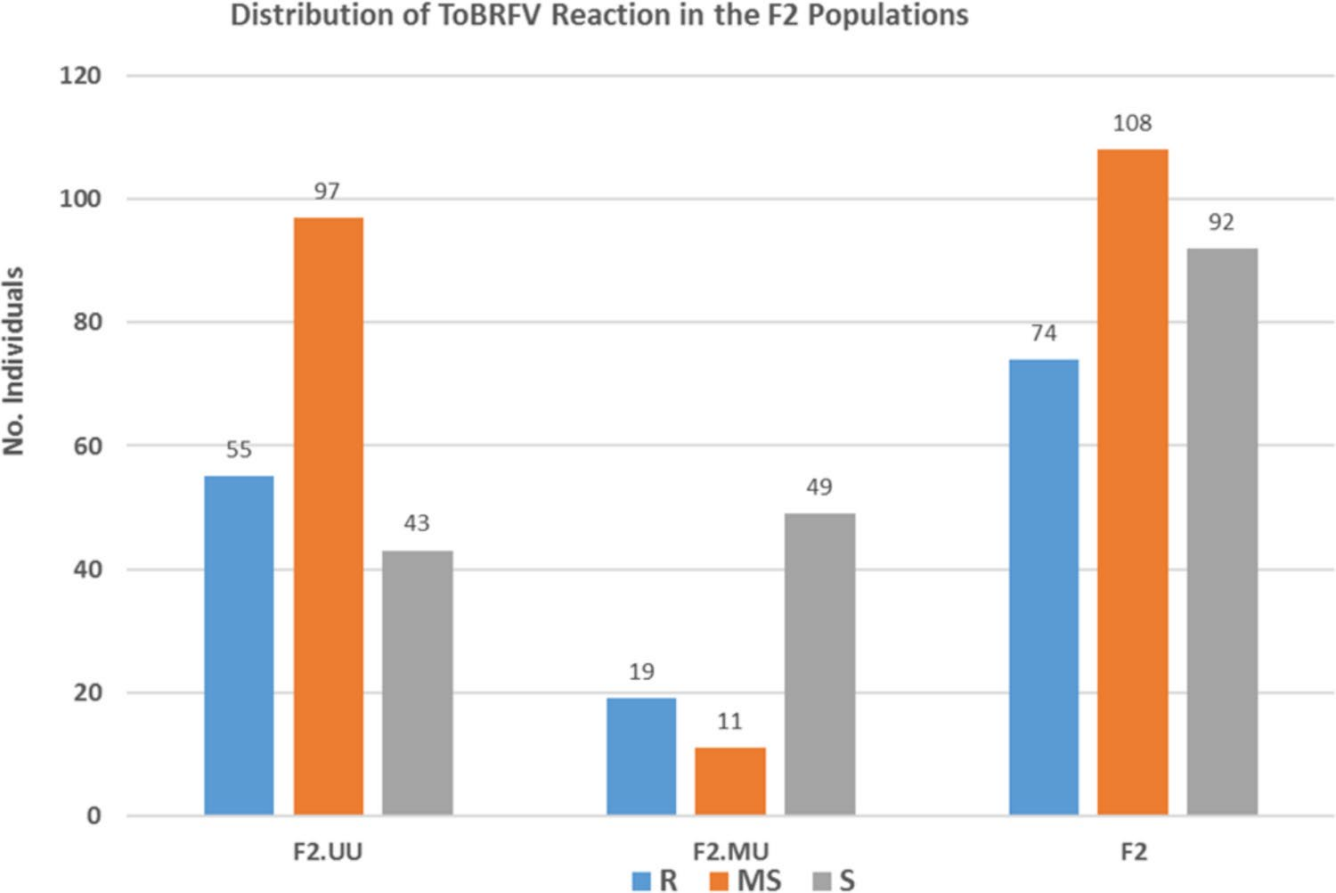
Distributions of resistance (R), intermediate susceptible (MS), and susceptible (S) individuals against tomato brown rugose fruit virus infection in the two individual F_2_ populations (F_2_.UU and F_2_.MU) and a combination of both F_2_ populations (F_2_)

The parent *S. pimpinellifolium* ‘USVL332’ exhibited resistance to ToBRFV infection, whereas the two other parents, *S. pimpinellifolium* ‘USVL333’ and *S. lycopersicum* ‘Moneymaker,’ were susceptible (Fig. 1). All F_1_ individuals from both populations, ‘USVL333’ x ‘USVL332’ (UU) and ‘Moneymaker’ x ‘USVL332’ (MU), were susceptible to ToBRFV infection (Fig. 1), indicating that resistance in ‘USVL332’ was recessive. The resistance segregation ratio in the F_2_ population followed a 1 resistant (R):3 susceptible (S) (MS + S) ratio, with *P* = 0.30 for the intraspecific cross (UU) and *P* = 0.85 for the interspecific cross (MU). Similarly, the combined F_2_ populations also conformed to a 1 R:3 S (MS + S) ratio, with *P* = 0.44 (Table 1 and Fig. 2), confirming the recessive inheritance of resistance in USVL332*.*

**Table 1:** Chi-square test for segregation ratios of resistant and susceptible plants in F_2_ populations

F2 population	R	MS	S	Total	R	MS + S	E(R)	E(S)	χ^2^ [1R:3(M + S)]	*P*-value
F2.UU	55	97	43	195	55	140	48.75	146.25	1.068	0.30
F2.MU	19	11	49	79	19	60	19.75	59.25	0.038	0.85
F2	74	108	92	274	74	200	68.5	205.5	0.589	0.44

### Population structure and principal component analysis

**Fig. 3:**
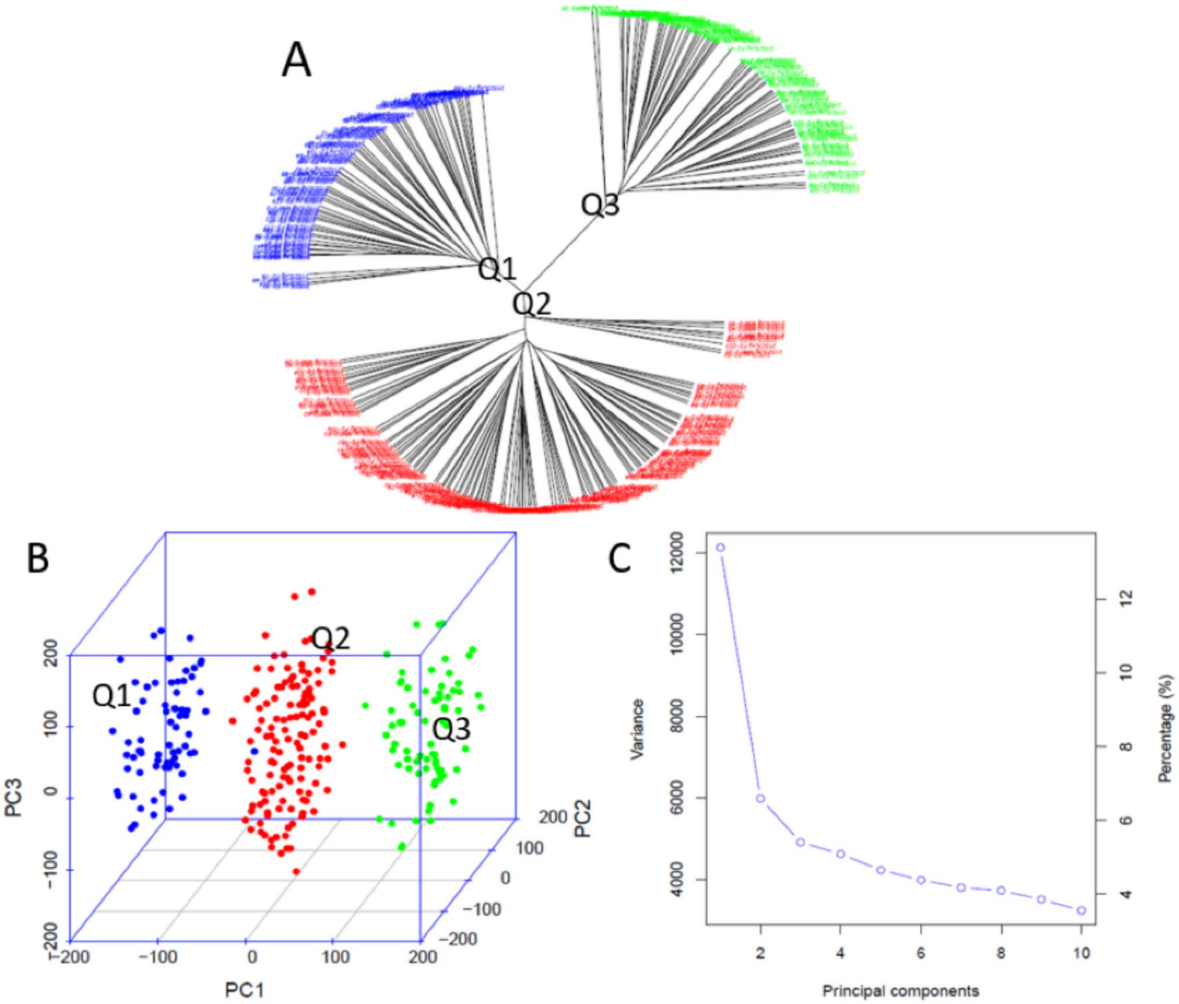
Population genetic diversity analysis of two F2 populations consisting of 274 individuals. **A** Unrooted phylogenetic tree constructed using the neighbor-joining (NJ) method, showing three sub-populations; **B** 3D graphical plot of principal component analysis (PCA), illustrating the three sub-populations; and **C** PCA eigenvalue plot generated using GAPIT 3, showing that the top three principal components (PCA = 3) explain majority of the variation among the 274 F2 individuals

Population genetic diversity analysis was conducted on the two F_2_ populations, consisting of 274 individuals. Phylogenetic trees were constructed using the neighbor-joining (NJ) method in GAPIT 3, resulting in an unrooted tree (Fig. 3A) displaying three distinct sub-populations; a 3D graphical plot of principal component analysis (PCA) (Fig. 3B) illustrating the three sub-populations; and a PCA eigenvalue plot (Fig. 3C), generated using GAPIT 3, indicating that the first three principal components (PCs) explain the majority of the variation among the 274 F_2_ individuals. These plots and phylogenetic trees revealed the presence of three distinct clusters within the two combined F_2_ populations. The PCA data for the three sub-populations were subsequently used as a Q-matrix in the GWAS analysis to identify SNP markers associated with ToBRFV resistance.

Among the three clusters (sub-populations), Q1 consisted of 71 individuals (25.9% of the entire population), including 28 from F2.MU (35.4% of F2.MU) and 43 from F2.UU (22.1% of F2.UU). Q2 included 131 individuals (47.8%), comprising 35 from F2.MU (44.3%) and 96 from F2.UU (49.2%). Q3 comprised 72 individuals (26.3%), with 16 from F2.MU (20.3%) and 56 from F2.UU (28.7%) (Table S1a, S1b, S1c-Fig). These results indicate the presence of three distinct sub-populations (clusters) within each of the F2 populations (F2.MU and F2.UU), as well as in the combined population, which supports the suitability of this material for GWAS.

### Association study

**Fig. 4:**
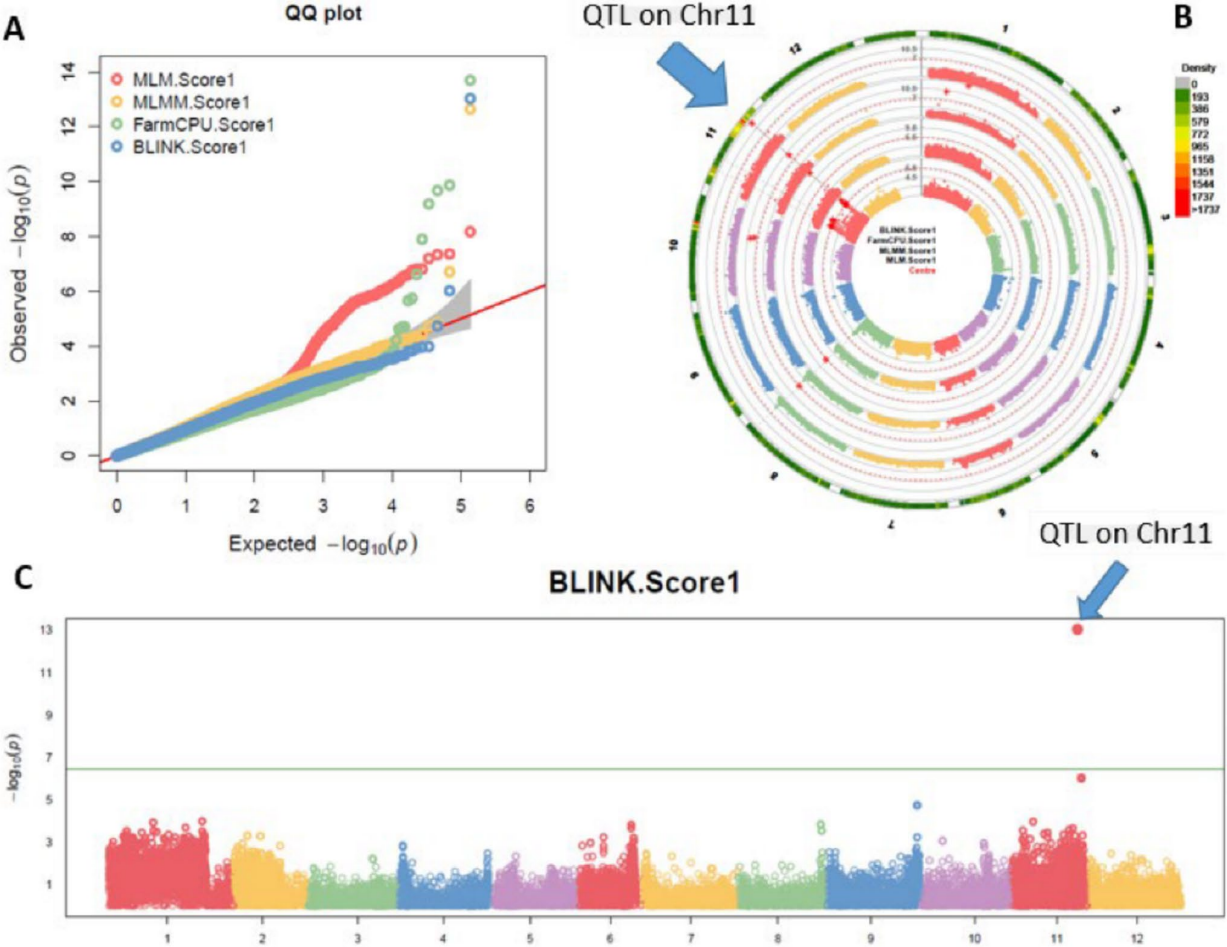
Genome wide association study (GWAS) for ToBRFV resistance in *Solanum pimpinellifolium* was conducted. Four models: MLM, MLMM, FarmCPU, and Blink in GAPIT 3 were used to generate a QQ-plot (**A**) and Manhattan plots (**B**). A BLINK Manhattan plot (**C**) revealed SNP markers significantly associated with the ToBRFV resistance on chromosome 11

Association analysis for ToBRFV resistance was conducted using four models: MLM, MLMM, FarmCPU, and Blink in GAPIT 3, and three models: SMR, GLM, and MLM in TASSEL 5. The observed versus expected LOD [-log10(p)] distributions in QQ-plots showed a significant divergence from the expected distribution across the four models (MLM, MLMM, FarmCPU, and Blink) (Fig. 4A), indicating the presence of SNPs associated with ToBRFV resistance in ‘USVL332’ within the F_2_ populations. The multiple Manhattan plots generated from the four models (Fig. 4B) identified significant SNPs on chromosome 11 with LOD values greater than 6.44 (the significance threshold), suggesting their association with ToBRFV resistance. Additionally, the multiple Manhattan and QQ-plots for ToBRFV resistance based on the four models are provided in Supplementary Figure S1. A Manhattan plot generated using the Blink model highlights a highly significant associated SNP marker (Fig. 4C).

The QQ-plots and Manhattan plots generated using the three models in TASSEL 5 (Supplementary Fig. S2) showed similar trends to those from GAPIT 3 for ToBRFV resistance, with numerous significant SNP markers observed on chromosome 11. This suggests the presence of QTLs for ToBRFV resistance on chromosome 11.

Across the four models in GAPIT 3 and three models in TASSEL 5, five SNPs were identified as significantly associated with ToBRFV resistance. These SNPs, located within a ~ 178 kbp region spanning from 46,825,788 bp to 47,003,077 bp on chromosome 11, had LOD [-log(P)] values exceeding 6.44 in three or more of the seven models used (Table 2 and Fig. 4). In TASSEL 5, these SNPs had LOD values exceeding 12 in SMR, greater than 9 in GLM, and above 5.8 in MLM. Similarly, in GAPIT 3, the LOD values exceeded 6.5 in MLM (Table 2). Among these five SNPs, SL4.0ch11_46825788 showed the strongest association, with LOD > 6.44 in six out of seven models, including 13.03 in BLINK and 9.19 in FarmCPU. SL4.0ch11_46847421 had LOD > 6.44 in five models, including a value of 12.65 in MLMM. SL4.0ch11_46850215 and SL4.0ch11_46850230 each had LOD > 6.44 in three models, with LOD values close to 6.0 in TASSEL MLM. SL4.0ch11_47003077 had LOD > 6.44 in four models (Table 2). These SNPs ranked in significance as follows: SL4.0ch11_46825788 > SL4.0ch11_46847421 > SL4.0ch11_47003077 > SL4.0ch11_46850215 > SL4.0ch11_46850230. The R-squared values for these SNPs ranged from 10.89% for SL4.0ch11_46850230 in TASSEL MLM to 24.75% for SL4.0ch11_46847421 in SMR, with an average of 16.7% (Table 2). These results confirm the presence of a QTL on chromosome 11 associated with ToBRFV resistance.

**Table 2:** SNP markers associated with ToBRFV resistance based on seven models

SNP	Chr	Pos (bp)	MAF%	LOD[-Log(-value)] in GAPIT3				LOD[-Log(-value)] in Tassel 5			%R-square in Tassel 5		
				MLM	MLMM	FarmCPU	BLINK	SMR	GLM	MLM	SMR	GLM	MLM
SL4.0ch11_46825788	11	46,825,788	14.8	7.37	1.14	9.19	13.03	16.20	12.30	7.56	24.54	15.80	13.93
SL4.0ch11_46847421	11	46,847,421	15.0	8.17	12.65	0.55	2.03	16.36	12.99	8.48	24.75	16.49	15.85
SL4.0ch11_46850215	11	46,850,215	15.3	6.81	0.59	0.26	0.75	13.32	10.03	6.19	20.87	13.23	11.32
SL4.0ch11_46850230	11	46,850,230	15.1	6.61	0.33	0.08	0.33	12.46	9.32	5.84	19.94	12.53	10.89
SL4.0ch11_47003077	11	47,003,077	13.5	6.50	0.32	0.34	0.93	15.25	11.12	6.78	23.21	14.25	12.42

### Genetic mapping and QTL analysis

We attempted to create SNP genetic maps using JoinMap 3 and MSTMap from the individual UU, MU, and combined F_2_ populations. However, the genetic maps generated for each chromosome did not align with the physical maps, as over 90% of the SNPs did not exhibit a 1:2:1 segregation pattern. From the GWAS analysis, only chromosome 11 showed a significant QTL region associated with ToBRFV resistance. The 26,357 SNPs on chromosome 11 were too dense to perform effective QTL mapping with the small sample sizes of the individual F_2_ populations (UU, MU, or combined, consisting of 195, 79, or 274 individuals, respectively). Therefore, we selected 51 SNPs within the QTL region identified by GWAS to create a linkage map for further QTL analysis of ToBRFV resistance.

**Fig. 5:**
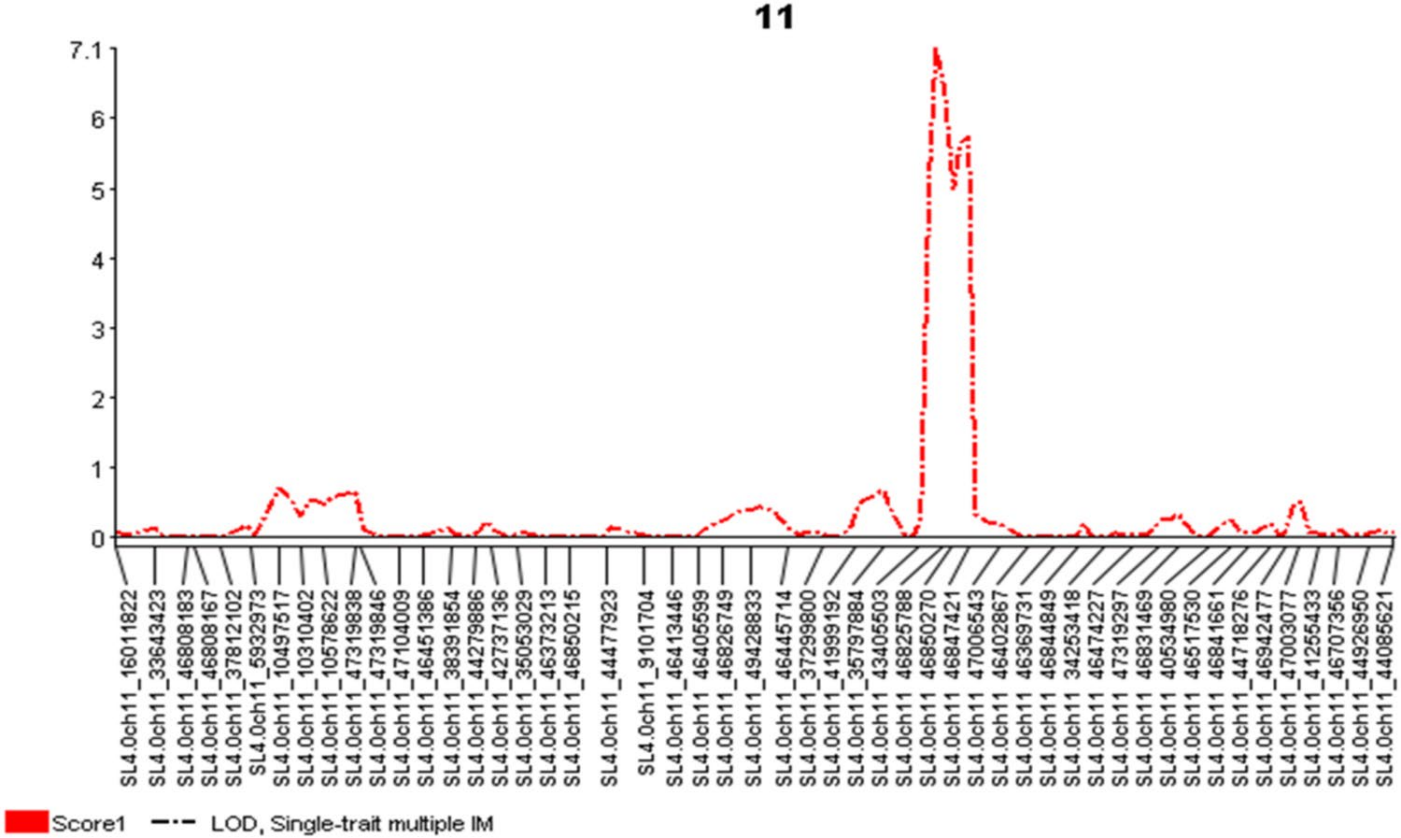
The genetic map at the QTL region with the viewable SNPs on chromosome 11 created by single-trait multiple interval mapping (SMIM) for ToBRFV resistance, where x-axis presents genetic map with SNP markers, and y-axis presents LOD value

QTL mapping using SMR, SMIM (Fig. 5), and BIM (Supplementary Fig. S3) in Qgene revealed a peak on chromosome 11 for ToBRFV resistance. Three SNPs, SL4.0ch11_46825788, SL4.0ch11_46850270, and SL4.0ch11_46847421, were tightly linked to ToBRFV resistance, with high LOD values of 14.25 in SMR, 7.01 in SMIM, and a posterior value of 0.88 in BIM (Table 3). These results indicate that a QTL for ToBRFV resistance exists in the SL4.0ch11_46825788—SL4.0ch11_46850270—SL4.0ch11_46847421 region of chromosome 11. The R^2^ values ranged from 5.5% to 35.5% using the single-trait multiple interval mapping (SMIM) model and from 38.2% to 56.4% for key SNPs (Table 3). These findings support the presence of a major-effect QTL in this region.

**Table 3:** QTL and linked SNP markers for ToBRFV resistance based on three models in Qgene

Mapping Model	SNP	Pos (cM)	Additive effect	LOD	%R^2^
Singe marker regression (SMR)	SL4.0ch11_46825788	214.9	2.82	13.95	55.7
	SL4.0ch11_46850270	217	2.78	13.33	54
	SL4.0ch11_46847421	221.6	2.81	14.25	56.4
	SL4.0ch11_47006543	229.3	2.33	8.26	38.2
Single-trait multiple interval mapping (SMIM)	SL4.0ch11_46825788	216–220	0.42–3.16	3.93–7.01	5.5–33.5
	SL4.0ch11_46850270				
	SL4.0ch11_46847421				

Mapping Model	SNP	Pos (cM)	Additive effect	Posterior
Single-trait Bayesian interval mapping (BIM)	SL4.0ch11_46825788	216–218	2.13–2.53	0.27–0.88
	SL4.0ch11_46850270			
	SL4.0ch11_46847421			

### Candidate gene identification/detection

A total of 17 genes were identified within the QTL region between 46.7 Mbp and 47.1 Mbp on chromosome 11 (Supplementary Table S2). Among these 17 genes, two disease resistance gene analogs were identified as potential candidates for ToBRFV resistance in ‘USVL332.’ These include Solyc11g062150, a TIR-NBS-LRR resistance protein (Toll-Interleukin receptor), located at 46,961,662 bp–46,964,437 bp, and Solyc11g062180, a disease resistance protein with leucine-rich repeat (LRR) domains, located at 46,983,996 bp–46,984,571 bp on chromosome 11 (SL4.0ch11). In addition, Solyc11g062160 (46,979,044–46,979,646 bp), encoding an endonuclease/exonuclease/phosphatase, was located within this region. These enzyme families are known to contribute to plant immune responses by participating in DNA replication and repair, while phosphatases modulate intracellular signaling pathways during stress (Schweighofer et al. 2004). Furthermore, Solyc11g062060 (46,837,096–46,839,707 bp), encoding a C2H2-type zinc finger protein, belongs to a gene family implicated in the transcriptional regulation of defense-related genes (Huang et al. 2024). These observations suggest that allelic variation in these candidate genes may underlie the resistance phenotype observed in ‘USVL332.’ Resequencing-based SNP and InDel analyses are underway, and functional validation is in progress to confirm their roles in ToBRFV resistance.

## Discussion

### ToBRFV resistance in *S. pimpinellifolium*

A diverse range of *Solanum* species, including tomato breeding lines and wild relatives, have been identified as resistance or tolerance to ToBRFV (Salem et al. 2023). Apart from one resistance source in *S. lycopersicum* and another tolerant *S. pimpinellifolium* (Zinger et al. 2021), most other resistant or tolerant materials have not been genetically characterized or made publicly available (Salem et al. 2023). A recent study demonstrated that the resistance to ToBRFV in *S. lycopersicum* involves an interaction of the *Tm-1* gene and an unknown locus on chromosome 11 (Zinger et al. 2025). In this study, we used the resistant *S. pimpinellifolium* ‘USVL332,’ which was previously identified in our earlier work (Jaiswal et al. 2024). We developed two F_2_ populations (a total of 274 individuals) from two crosses: one an intraspecific cross between *S. pimpinellifolium* ‘USVL332’ and ‘USVL333’ (195 F_2_ plants), and another an interspecific cross between *S. pimpinellifolium* ‘USVL332’ and *S. lycopersicum* ‘Moneymaker’ (79 F_2_ plants). Both ‘USVL332’ and ‘USVL333’ were derived through a single-seed descent for three generations (S3) from PI 390717 and PI 390718, respectively. These two *S. pimpinellifolium* accessions were originally collected from Peru but exhibited distinct responses to ToBRFV infection. While PI 390717 was resistant, PI 390718 was susceptible to ToBRFV infection in our germplasm screening (Jaiswal et al. 2024).

Upon confirming their respective resistance or susceptibility to ToBRFV, two parental lines, ‘USVL332’ and ‘USVL333,’ were crossed. The F_2_ populations generated from this intraspecific cross between the two *S. pimpinellifolium* lines were selected for QTL analysis due to their similar genetic background, with a major difference in phenotype regarding their resistance or susceptibility to ToBRFV infection. Resistance in ‘USVL332’ was characterized by low to non-detectable levels of virus titer in infected plants, determined by ELISA and qRT-PCR testing (Jaiswal et al. 2024). In contrast, ‘USVL333’ exhibited mosaic and mottling symptoms on infected plants, with a high virus titer, similar to that of the susceptible tomato control ‘Moneymaker’ (Jaiswal et al. 2024). However, the resistance to ToBRFV in ‘USVL332’ is not complete immunity. Nevertheless, its high resistance to ToBRFV and the ease of cross-pollination with *S. lycopersicum* make this line an attractive source of genetic resistance for incorporation into common tomato cultivars and breeding lines aimed at improving ToBRFV resistance.

### QTL and association mapping of ToBRFV resistance

QTL mapping is based on the association of phenotypic and genotypic data to map QTLs to chromosomes in segregating genetic populations (F_2_, F_2:3_, RIL) using statistical models to tag major or minor genes/alleles in crops. In the present study, JoinMap 4 (Van Ooijen 2006) and MSTmap (Wu et al. 2008) were used to create genetic linkage maps in two F_2_ populations of 274 individuals: One derived from an intraspecific cross between *S. pimpinellif*olium ‘USVL332’ and ‘USVL333,’ and the other from an interspecific cross between *S. pimpinellifolium* ‘USVL332’ and *S. lycopersicum* ‘Moneymaker.’ A total of 136,357 SNPs were used to construct the genetic maps, incorporating both genetic and physical distances. However, the SNP orders did not align well between the genetic and physical locations in each chromosome. This discrepancy may have been due to the small sizes of the mapping populations, with 195 F_2_ individuals from UU and 79 F_2_ from MU.

To overcome the genetic order errors in QTL mapping, we performed a GWAS for ToBRFV resistance in the two F_2_ populations. Using four models in GAPIT 3 and three models in TASSEL 5, we generated QQ-plots and Manhattan plots, which identified a peak on chromosome 11 for ToBRFV resistance (Fig. 5). Three SNPs, SL4.0ch11_46825788, SL4.0ch11_46850270, and SL4.0ch11_46847421, showed significant associations with ToBRFV resistance, with high LOD values (Table 3), indicating the presence of a QTL in this region of chromosome 11. The identification of a major QTL on chromosome 11 for ToBRFV resistance is consistent with the findings of a previous study on *S. lycopersicum* (Zinger et al. 2021).

### Candidate gene for ToBRFV resistance

In the QTL region between 46.7 Mbp and 47.1 Mbp on chromosome 11, a total of 17 genes were identified (Table S2). Among them, two disease resistance gene analogs were of particular interest: Solyc11g062150**,** a TIR-NBS-LRR resistance protein (Toll-Interleukin receptor), and Solyc11g062180**,** a disease resistance protein with a leucine-rich repeat (LRR). Further characterization of these genes is necessary to determine if they are candidate genes for ToBRFV resistance in *S. pimpinellifolium* ‘USVL332.’

## Conclusion

In this study, a QTL region for ToBRFV resistance was identified in two F_2_ populations derived from *S. pimpinellifolium* ‘USVL333’ × ‘USVL332’ and ‘Moneymaker’ × ‘USVL332.’ The QTL was located in the approximately 46.84 Mbp region on chromosome 11, with three SNP markers showing the highest LOD values (> 15) in association with the ToBRFV resistance locus. The disease gene analogs Solyc11g062150 (TIR-NBS-LRR resistance protein, Toll-Interleukin receptor) and Solyc11g062180 (disease resistance protein, leucine-rich repeat) on chromosome 11 (SL4.0ch11) were recognized as potential candidates for ToBRFV resistance in *S. pimpinellifolium*. The three identified SNPs could be further explored for the development of ToBRFV-resistant cultivars through marker-assisted selection, while the candidate disease resistance genes could serve as targets for functional analysis.

## Supplementary Information

Below is the link to the electronic supplementary material.

Supplementary file1 (XLSX 1300 KB)

## Data Availability

The datasets presented in this study are available in Tables, Figures, Supplementary Tables, and Supplementary Figures. The SNP data are available in FigShare https://doi.org/10.6084/m9.figshare.25345042.v1. The accession number(s) used in this study can be found in the article/Supplementary Material.
